# High-throughput sequencing yields a complete mitochondrial genome of the rice thrips, *Stenchaetothrips biformis* (Thysanoptera: Thripidae)

**DOI:** 10.1080/23802359.2023.2169572

**Published:** 2023-02-05

**Authors:** Qing-Ling Hu, Zhuang-Xin Ye, Chuan-Xi Zhang

**Affiliations:** a Institute of Insect Science, Zhejiang University, Hangzhou, China; b State Key Laboratory for Managing Biotic and Chemical Threats to the Quality and Safety of Agro-Products, Key Laboratory of Biotechnology in Plant Protection of Ministry of Agriculture and Zhejiang Province, Institute of Plant Virology, Ningbo University, Ningbo, China

**Keywords:** Mitochondrial, *Stenchaetothrips biformis*, thysanoptera

## Abstract

Rice thrips, *Stenchaetothrips biformis* (Bagnall, 1913), are one of the destructive pests of rice. Here, the complete mitochondrial genome of *S. biformis* was sequenced using high-throughput sequencing. The mitogenome is 15,359 bp long with an A + T content of 76.94%, which contains 13 protein-coding genes (PCGs), 22 transfer RNA (tRNAs), 2 ribosomal RNA genes (rRNAs) and 2 putative control regions (CRs). The phylogenetic analysis showed that *S. biformis* is closely related to *Thrips imaginis* and *Thrips palmi*. This new mitochondrial genome data can be better used to provide a basis for studies of the mitochondrial evolution of Thysanoptera.

## Introduction

In eukaryotic cells, the genomes of mitochondria contain tRNAs-, rRNAs-, and protein-coding genes, whose functions appear to be universally conserved, however, the gene content, arrangement expression, and even the genomic size exhibit remarkable variation (Gray 2012). In insects, the mitochondrial genome is the most extensively researched genomic system and is used as a popular molecular marker for phylogenetic inference, identification of species origin, analysis of population structure and dynamics, molecular evolution, and so on (Cameron 2014).

Thysanoptera contains more than 5500 species, most of which are tiny and linear, a large of these species are phytophagous, and even some species are globally important crop pests. More and more thrips’ mitochondria genomics are sequenced and reported, in which gene rearrangement is characteristic, such as the plague thrips, *Thrips imagines*; the flower thrips *Frankliniella intonsa*; the western flower thrips, *Frankliniella occidentalis* (Shao and Barker 2003; Yan et al. 2012, 2014). The rice thrip, *Stenchaetothrips biformis*, is one of the most important rice pests in Europe, South America, and Asia, which attacks in the seedling stage of rice and causes huge losses (Nugaliyadde and Heinrichs 1984). However, the full mitochondrial genome is still unknown, which limits this species’ phylogeny study, species diagnostics, biogeography study, and so on. In this study, we got the complete mitochondrial genome sequence through high-throughput sequencing.

## Materials and methods

### Ethics statement

The program of insect collection and experiment in the article has passed the ethical review of Animal and plant Ethics Committee of Ningbo University.

### Sample collection

Living *Stenchaetothrips biformis* individuals were collected from rice seedlings in NingBo (29°54′N, 121°38′E), Zhejiang, China. The specimen and DNA were deposited at the Institute of Plant Virology of Ningbo University (Specimen ID: NB-20200809; http://ipv.nbu.edu.cn) by Pro. Chuan-Xi Zhang (chxzhang@zju.edu.cn).

### Mitochondrial genome assembly and annotation

Genomic DNA was isolated by Wizard® Genomic DNA Purification Kit according to the manufacturer’s instructions. The complete mitochondrial genome of *S. biformis* (GenBank Accession number: ON653412) was sequenced using Illumina HiSeq 4000, and A de novo assembler soft NOVOPlasty was used to do the assembly (Dierckxsens et al. 2017). The protein-coding genes (PCGs), tRNAs, rRNAs, and 2 control regions (CRs) were analyzed by MITOS2 online webserver (Bernt et al. 2013).

### Phylogenetic analysis

Sequences used in the phylogenetic analysis were obtained from the NCBI GenBank database, the “GTR + G4” model was employed to construct a phylogenetic tree in Software Raxml-ng with 1000 bootstrap replications (Kozlov et al. 2019).

## Results

**Figure 1.**
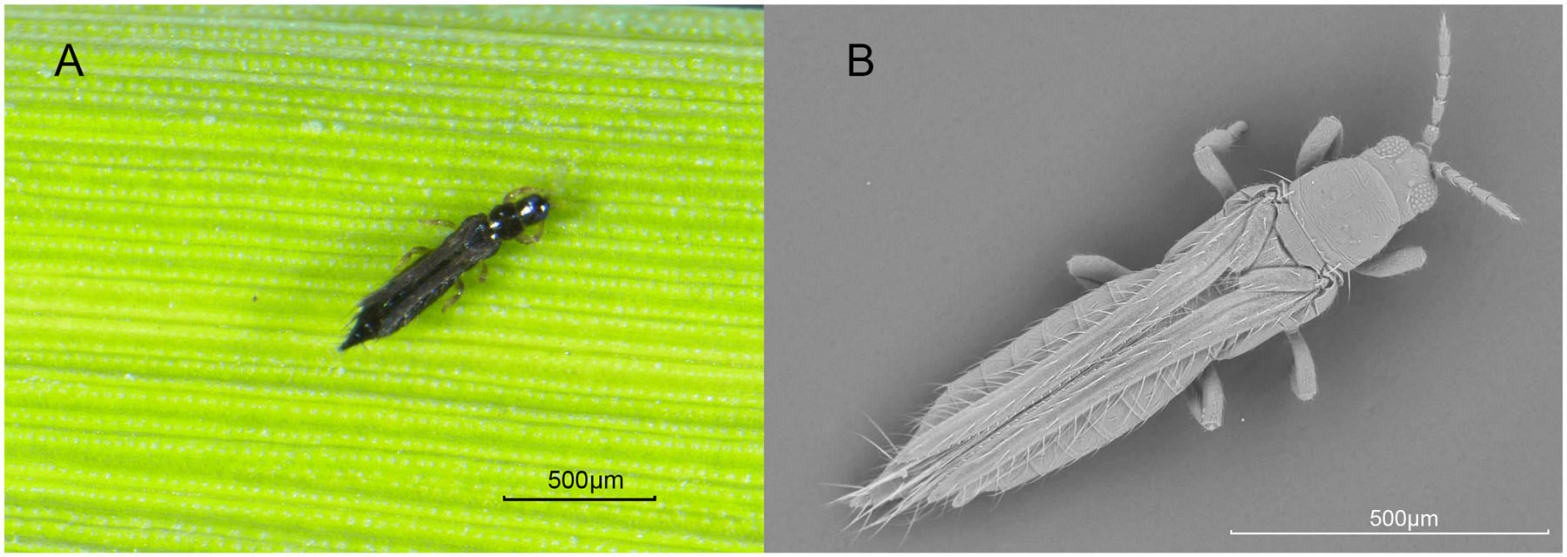
The female adult of *S. biformis*. These pictures were taken by us using (A) color stereoscope microscope (B) and grey scanning electron microscope, respectively. Scale bar: 500 µm.

**Figure 2.**
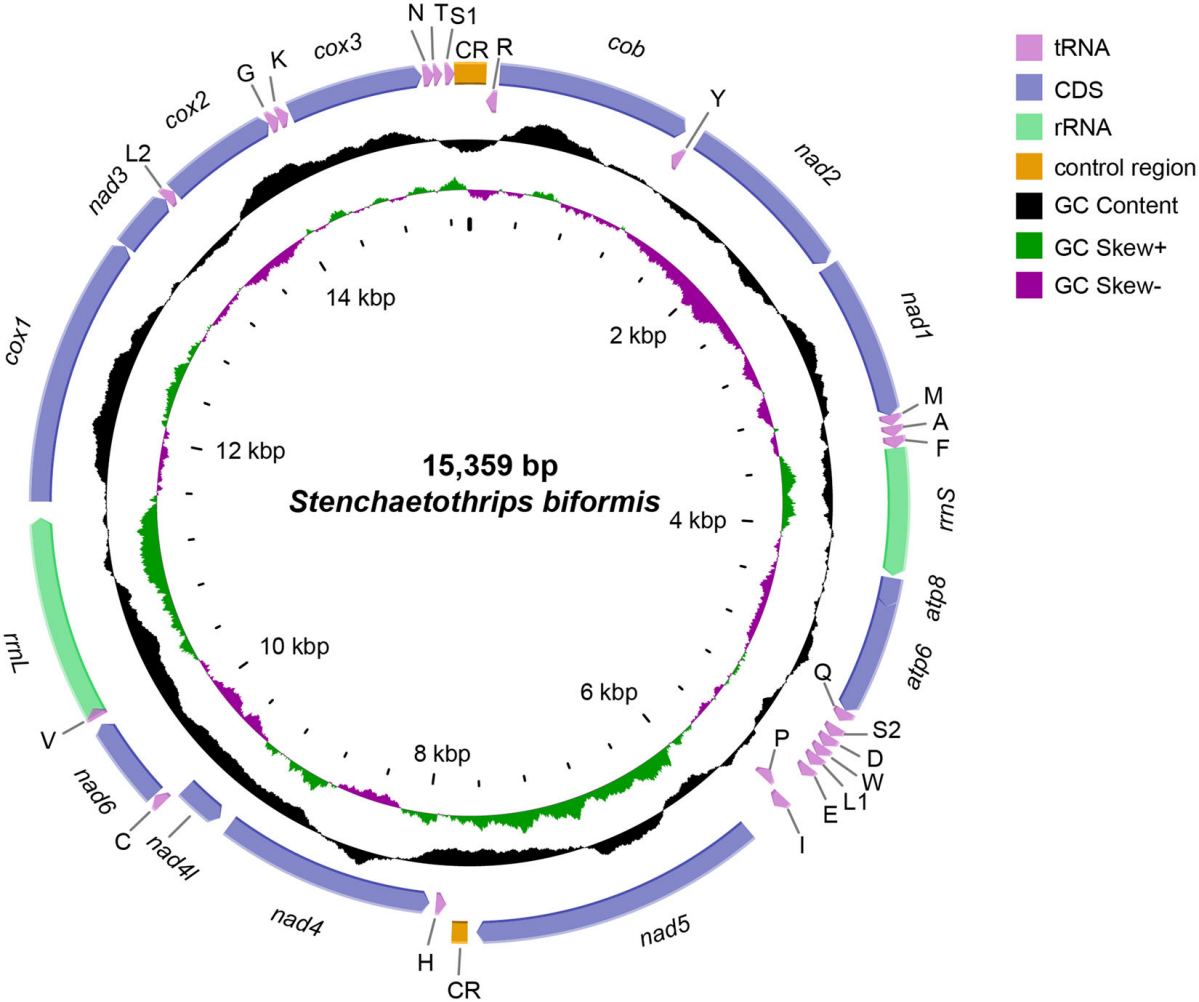
The circular complete mitochondrial genome map of *S. biformis*. The gene transcriptional direction as indicated by the arrow direction. This figure was plotted by CGView online server (https://proksee.ca/).

The rice thrips, *Stenchaetothrips biformis*, is tiny and linear, about 1–1.3 mm length (Figure 1). The full length of the complete mitochondrial genome of *S. biformis* is 15,359 bp, the content of A + T is 76.94% with A (39.31%) and T (37.63%), the content of C is 12.61% and G is 10.45%, showing an obvious bias toward A and T (Figure 2). Totally, 37 genes were encoded, including 13 PCGs, 22 tRNAs and 2 rRNAs. The 13 PCGs encoded *cob*, *nad2*, *nad1*, *atp8*, *atp6*, *nad5*, *nad4*, *nad41*, *nad6*, *cox1*, *nad3*, *cox2*, *cox3* respectively, in which only *atp8* and *nad3* use an incomplete stop codon “T–." The two rRNAs, *rrnS* and *rrnL*, were 730 bp and 1,177 bp in length, respectively.

**Figure 3.**
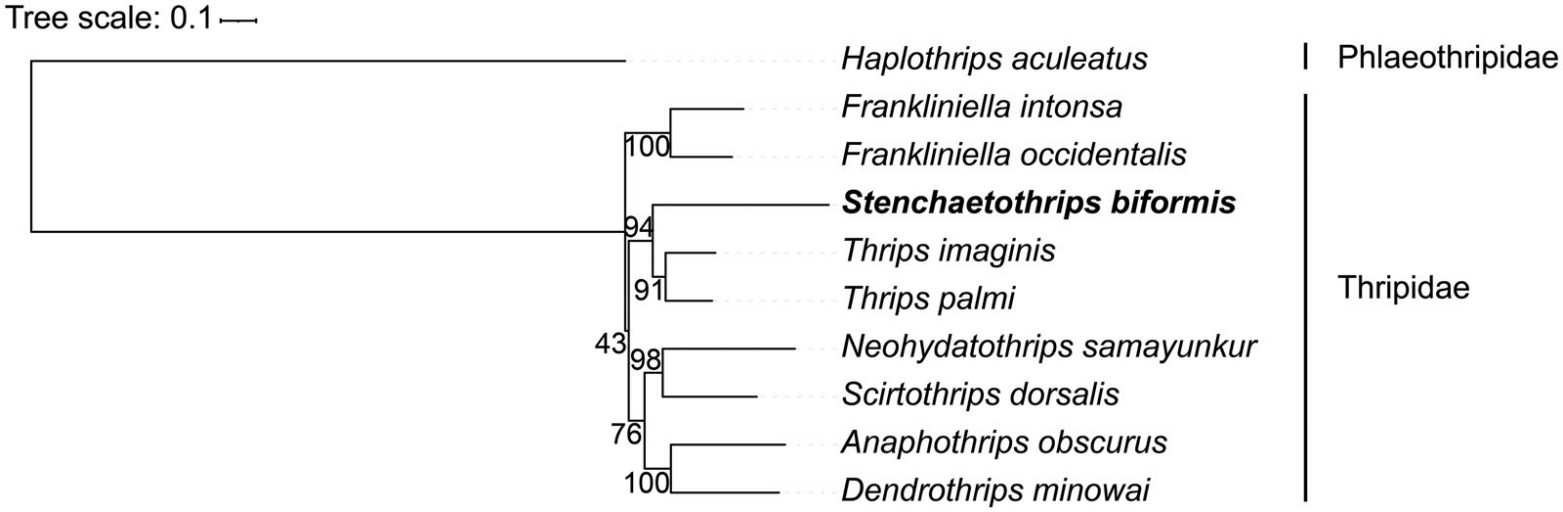
Phylogeny of ten species of Thripidae based on the protein-coding genes. The following sequences were used: Haplothrips aculeatus, NC 027488.1; Frankliniella intonsa, NC 021378.1; Frankliniella occidentalis, NC 018370.1; Stenchaetothrips biformis, ON653412.2; Thrips imagines, NC 004371.1; Thrips palmi, NC 039437.1; Neohydatothrips samayunkur, NC 039942.1; Scirtothrips dorsalis, NC 025241.1; Anaphothrips obscurus, NC 035510.1; Dendrothrips minowai, NC 037839.1. The support values are shown next to the nodes.

To assess the mitochondrial sequence authenticity of *S. biformis* and its phylogenetic position, we selected other 9 thysanopteran species to reconstruct phylogenetic tree using Raxml-ng (Huelsenbeck and Ronquist 2001; Shao and Barker 2003; Yan et al. 2012, 2014; Liu et al. 2017; Chakraborty et al. 2018; Chen et al. 2018; Kumar et al. 2019). The analysis of phylogeny showed that *S. biformis* is closely related to *Thrips imaginis* and *Thrips palmi* (Figure 3).

## Discussion and conclusion

In summary, we assembled the mitochondrial genome of rice thrips, *S. biformis*, 15,359 bp in length, whose size is middle in Thripidae species, we also found 37 genes were coded and two control regions distributed on the genome. Thrips mitochondrial genomes are marked by high rates of gene rearrangement, our results could help to understand these phenomena. The complete mitochondrial genome of *S. biformis* can also be better used to identify the species of Thripidae and study the evolution of Thripidae.

## Data Availability

The genome sequence data that support the findings in this study are openly available in GenBank of NCBI at (https://www.ncbi.nlm.nih.gov/) under accession no. ON653412. The associated BioProject, SRA, and Bio-Sample numbers are PRJNA846937, SRR19576561, and SAMN28906286, respectively.
